# Toxin gene profiles, antibiotic resistance, and genetic diversity of *Clostridium perfringens* from food-producing animals: a whole-genome sequencing study with implications for food safety

**DOI:** 10.1016/j.crfs.2026.101460

**Published:** 2026-06-01

**Authors:** Shuangyu Xie, Simeng Liu, Stefan Schwarz, Hongfei Sun, Qiu Xu, Jiyun Chai, Longhua Lin, Susu Du, Jie Hou, Yongshan Song, Andrea Brenciani, Yao Zhu, Wanjiang Zhang

**Affiliations:** a State Key Laboratory of Animal Disease Control and Prevention, Harbin Veterinary Research Institute, Chinese Academy of Agricultural Sciences, Harbin, 150069, China; b Institute of Microbiology and Epizootics, Centre for Infection Medicine, School of Veterinary Medicine, Freie Universität Berlin, Berlin, 14163, Germany; c Veterinary Centre for Resistance Research (TZR), School of Veterinary Medicine, Freie Universität Berlin, Berlin, 14163, Germany; d Unit of Microbiology, Department of Biomedical Sciences and Public Health, Polytechnic University of Marche Medical School, Ancona, Italy

**Keywords:** *Clostridium perfringens*, Food safety, Toxin genes, Antimicrobial resistance, Defense systems, MLST

## Abstract

*Clostridium perfringens* is a significant zoonotic foodborne pathogen. To systematically assess the potential risks associated with food-producing animals as a reservoir of *C. perfringens* in the early stages of the food production chain, we conducted whole-genome sequencing (WGS) and phenotypic analysis of 91 clinical *C. perfringens* isolates collected from pigs, chickens, cows, ducks, and geese across different regions of China. The results revealed that the isolates harbored a rich repertoire of toxin genes, with 71.43% (65/91) carrying greater than or equal to 10 toxin genes. Besides the classic type A, type C, which causes animal enterotoxemia, was most prevalent in pigs (45.76%). Notably, the and necrotic B-like (NetB) toxin, typically associated with avian necrotic enteritis, was also detected in isolates from cows and geese, suggesting potential cross-host transmission of toxin types. Antimicrobial susceptibility testing revealed a severe resistance situation, particularly among porcine isolates, which showed the highest resistance rates to clindamycin, penicillin, and tetracycline, with widespread multidrug resistance (MDR). Genomic analysis further identified 14 types of antimicrobial resistance (AMR) genes. The tetracycline resistance gene *tetA*(P) had an extremely high carriage rate of 94.51%, and AMR genes were most enriched in porcine isolates. Multilocus sequence typing (MLST) identified 59 sequence types (STs), 42 of which were newly discovered, demonstrating high genetic diversity. Major clonal complexes (CCs) showed certain host and geographic clustering. Furthermore, while the restriction-modification (RM) system was present in all isolates, the distribution of other defense systems like CRISPR-Cas was strain-specific. This study revealed that *C. perfringens* from Chinese food-producing animals is characterized by high virulence, extensive antimicrobial resistance, and high genetic diversity. It highlighted that pigs may serve as a crucial reservoir and evolutionary hub for virulent MDR isolates, posing a continuous threat to food safety and public health, and underscored the necessity for enhanced monitoring at the farm level.

## Highlights

• The *C. perfringens* isolates from food-producing animals harbored abundant toxin and antimicrobial resistance genes.
• The isolates comprised forty-eight known defense systems.
• The ninety-one isolates were classified into fifty-nine distinct STs, of which 42 were newly discovered.
• ST39 (pig) and ST21 (chicken) were the predominant epidemic isolates.

## Graphical abstract

**Figure:**
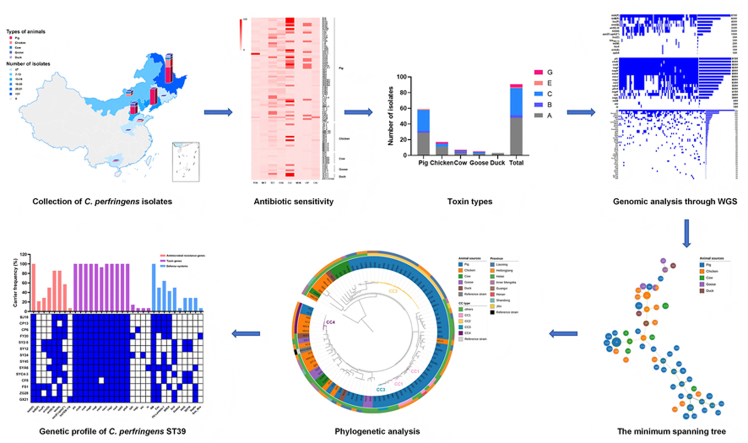

## 1 Introduction

*Clostridium perfringens* is one of the major foodborne pathogens, causing various intestinal diseases in humans and animals, such as food poisoning, gas gangrene, and necrotic enteritis. Its pathogenicity primarily depends on the production of protein toxins and enzymes (Rood et al., 2018). More than 20 toxins have been reported from *C. perfringens*, with different toxins causing distinct disease symptoms. Based on the production of six major toxins - alpha-toxin (*plc*), beta-toxin (*cpb*), epsilon-toxin (*etx*), iota-toxin (*iap*/*ibp*), *C. perfringens* enterotoxin (*cpe*), and necrotic enteritis B-like toxin (*netB*) - *C. perfringens* is classified into seven toxin types (A-G) (Kiu and Hall, 2018).

*C. perfringens* is ubiquitous in the environment and food due to its spore-forming ability, which allows survival under extreme or nutrient-deficient conditions (Hamza et al., 2018; Savard et al., 2026). Human ingestion of undercooked, contaminated animal-derived foods (e.g., pork, chicken and beef) can lead to rapid proliferation of pathogenic isolates and secretion of large amounts of toxins, causing intestinal infection and food poisoning (Mehdizadeh Gohari et al., 2021). *C. perfringens* is associated with human antibiotic-associated diarrhea (AAD) and necrotizing enterocolitis, among other diseases (Asha et al., 2006; Kiu et al., 2023). Reports of *C. perfringens*-caused foodborne disease outbreaks are gradually increasing worldwide, constituting a significant public health threat (Grass et al., 2013; Monma et al., 2015; Scallan et al., 2011).

Antimicrobial drugs are the primary method for treating bacterial diseases. However, long-term, frequent, and non-standardized use of antibiotics has led to the gradual development of antimicrobial resistance (AMR) (Gersema and Helling, 1986). The increasing prevalence of AMR is recognized as a global public health crisis. Livestock farming consumes over 70% of global antibiotics annually, with pig, chicken, and cattle production requiring more than 93,000 tons of antimicrobials to maintain animal health, making livestock manure an important reservoir for antibiotic resistance genes (ARGs) of human relevance (Mulchandani et al., 2023; Li et al., 2025; Van Boeckel et al., 2019). Although many countries have banned the use of antibiotics as feed additives, this reduction has been accompanied by an increasing number of *C. perfringens*-induced animal disease cases, with rising morbidity and mortality rates (B. Wang et al., 2021; Yan et al., 2024; Zhong et al., 2023).

With the increasing application of whole-genome sequencing (WGS), Multilocus Sequence Typing (MLST) based on WGS is becoming increasingly popular for epidemiological investigations and bacterial tracing. MLST is widely used to identify human, animal, and foodborne pathogens, to analyze bacterial population diversity and to investigate *C. perfringens* outbreaks (Abdel-Glil et al., 2021; Zhong et al., 2023). Bacterial defense systems, which protect against phage invasion and maintain genomic stability (e.g., restriction-modification systems and CRISPR) (Y. Wu et al., 2024), are as significant as virulence and resistance genes for bacterial genetic evolution (Soltani et al., 2025). However, analyses of defense systems in *C. perfringens* remain scarce. As a zoonotic foodborne pathogen, understanding the genomic characteristics of *C. perfringens* is crucial for public health. Although some studies have analyzed the genomes of *C. perfringens* in China, most focus on isolates from animals or humans in limited geographic areas (Fang et al., 2025; Xu et al., 2021). Systematic studies on the population genomic characteristics of *C. perfringens* from a wide range of provinces and various food-producing animals are still insufficient.

Therefore, in this study, we conducted a comprehensive genomic analysis of *C. perfringens* isolates from food-producing animals across different regions of China. We identified key genetic markers, including antibiotic resistance, toxin, and defense system-related genes, and analyzed genetic diversity and phylogenetic characteristics. These findings will enhance the understanding of the genetic diversity of *C. perfringens* and its ability to adapt to different environments, and provide data support for public food safety and public health risk assessment.

## 2 Materials and methods

### 2.1 Collection of *C. perfringens* isolates

A total of 91 *C. perfringens* isolates were obtained from 22 different cities or counties across eight key livestock-producing provinces in China between 2024 and 2025. The isolation sources included visceral organs, feces, and anal swabs collected from 349 samples of animals suspected of *C. perfringens* infection (Supplementary data 1, Fig. 1). Specifically, 59 isolates were derived from pigs, 17 from chickens, 7 from cows, 5 from geese, and 3 from ducks (Supplementary data 1, Fig. 1). The isolation and identification of these isolates were performed in accordance with the National Standard of the People's Republic of China, GB 4789.13-2012, with minor modifications. Briefly, bacterial cultures were inoculated onto tryptose sulfite cycloserine agar (Hope bio, China) and incubated anaerobically at 37 °C for 20-24 h. Uniform black colonies were picked and inoculated into fresh fluid thioglycollate medium (FTG) (Hope bio, China), followed by anaerobic incubation at 37 °C for 12 h. All isolates were stored at −80 °C in 25% (v/v) glycerol.

**Fig. 1:**
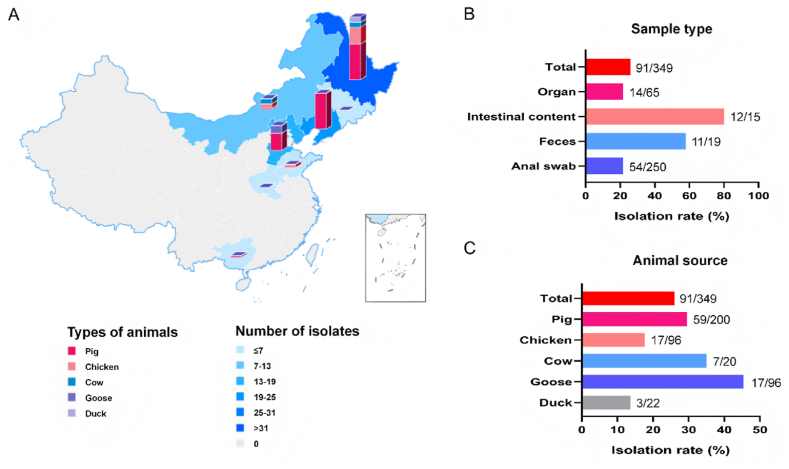
Geographic distribution of sampling locations and corresponding *C. perfringens* isolation rates. (A) Geographic distribution of sampling locations and animal sources. (B) Isolation rates of *C. perfringens* by sample type. (C) Isolation rates of *C. perfringens* by animal source.

### 2.2 DNA extraction and sequencing

WGS was performed to analyze the 91 *C. perfringens* isolates. Genomic DNA was extracted from the isolates using a TIANamp Bacteria DNA Kit (TIANGEN, China). Indexed DNA libraries were constructed with the TruSeq DNA PCR-free prep kit (Illumina, USA), and sequencing was carried out on the Illumina Novaseq Xplus system, generating 150-bp paired-end reads with a minimum coverage of 150-fold for each isolate. Raw reads were quality-filtered using Fastp software (v0.23.1) and subsequently assembled into contigs with SPAdes software (v3.11.1). Genome quality was assessed using CheckM software (v1.0.12), and only genomes with >95% completeness and <5% contamination were retained for further genomic analyses.

### 2.3 Identification of toxin, antimicrobial resistance, and defense genes

Toxin typing of each *C. perfringens* isolate was performed by detecting six major toxin genes (*plc, cpb, etx, ia, cpe,* and *netB*) via polymerase chain reaction (PCR) with primers designed from previously published sequences (Rood et al., 2018). Furthermore, toxin factor extraction and analysis were conducted using the Virulence Factor Database (VFDB; http://www.mgc.ac.cn/VFs/analyzer.htm) based on the WGS data of *C. perfringens*. The analysis targeted a panel of virulence factors, including sialidases, collagenases, phospholipase C, and other critical toxins, encoded by the genes *plc*, c*loSI*, *colA*, *nagH*, *nagI*, *nanH*, *nanJ*, *nagJ*, *nanI*, *cpb2*, *pfoA*, *nagK*, *cpb*, *nagL*, *etx*, *netB*, and *ia*. Antimicrobial resistance and defense genes were analyzed using the Center for Genomic Epidemiology (CGE; https://www.genomicepidemiology.org/services/) and DefenseFinder (https://defensefinder.mdmlab.fr/), respectively.

### 2.4 Antimicrobial susceptibility testing

Minimal inhibitory concentration (MIC) values were determined using the broth microdilution method as recommended by the Clinical and Laboratory Standards Institute (Clinical and Laboratory Standards Institute, 2018, 2020). The antibiotics used in this study (Shanghai yuanye Bio-Technology Co., Ltd, China) included penicillin (PEN), clindamycin (CLI), cefoxitin (FOX), chloramphenicol (CHL), meropenem (MEM), ciprofloxacin (CIP), tetracycline (TET), and metronidazole (MET). Serial two-fold dilutions of these antibiotics (0.125-128 μg/mL) were prepared in sterile 96-well microtiter plates. Each well was inoculated with a fresh culture of *C. perfringens* to achieve a final concentration of 1 × 10^6^ CFU/mL. The plates were then incubated at 37 °C for 48 h under anaerobic conditions. The reference strain *C. perfringens* ATCC 13124 was used as the quality control.

### 2.5 MLST and phylogenetic analysis

Sequence types (STs) were assigned to each *C. perfringens* genome based on the sequences of eight highly conserved housekeeping genes (*colA, groEL, sodA, plc, gyrB, sigK, pgk*, and *nadA*) sourced from the Institute Pasteur MLST scheme (https://bigsdb.pasteur.fr/). Novel allele sequences and STs were submitted to the PubMLST database (https://pubmlst.org/) for designation. Clonal complexes (CCs) were defined as groups of independent isolates sharing identical alleles at seven or more of the eight loci, with each CC assigned an arbitrary number. A minimum-spanning tree based on STs was generated using the PHYLOViZ online platform (www.phyloviz.net). Furthermore, a whole-genome phylogeny was constructed using Parsnp (https://github.com/marbl/parsnp), and the resulting phylogenetic tree was visualized and edited using the iTOL online platform (https://itol.embl.de/).

## 3 Results

### 3.1 *C. perfringens* isolates harbor a high abundance of toxin genes

A total of 91 *C. perfringens* isolates were recovered from 349 samples, yielding an overall isolation rate of 26.07%. By sample type, the highest isolation rate was observed in intestinal content (80.00%, 12/15), followed by feces (57.89%, 11/19), anal swabs (21.6%, 54/250), and organ samples (21.54%, 14/65). By animal sources, the isolation rate was highest in geese (45.45%, 5/11), followed by cattle (35.00%, 7/20), pigs (29.50%, 59/200), chickens (17.71%, 17/96), and ducks (13.64%, 3/22) (Fig. 1).

**Fig. 2:**
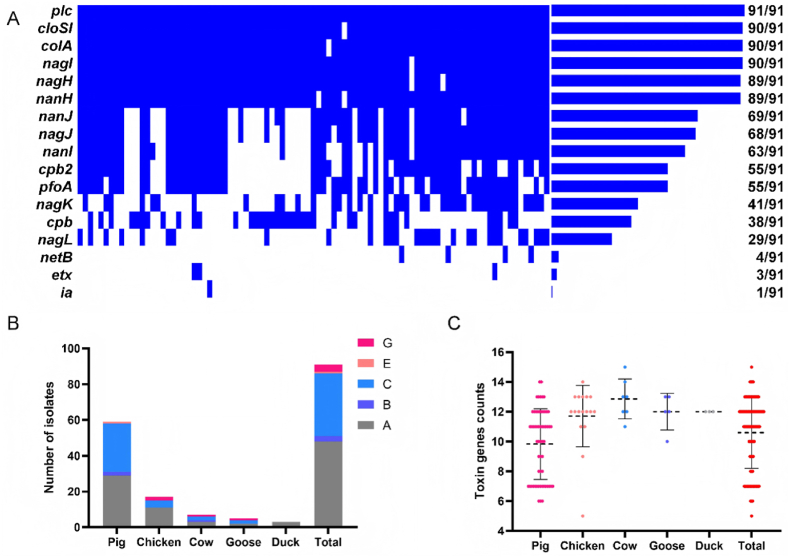
Toxin gene types in the *C. perfringens* isolates. (A) Toxin genes profile of the *C. perfringens* isolates. (B) Toxin types of the *C. perfringens* from different animal sources. (C) Numbers of toxin genes in the *C. perfringens* isolates from different animal sources.

The pathogenicity of *C. perfringens* is associated with its toxin-producing ability. Therefore, we classified the isolates into toxin types by examining genes related to the six major toxins involved in typing (*plc*, *cpb*, *etx*, *ia*, *cpe*, and *netB*) (Fig. 2, Table S1). The majority of isolates (48/91, 52.75%) were identified as *C. perfringens* type A. Notably, *C. perfringens* type C was most common in pigs (27/59, 45.76%) and was also detected in chickens, cows, and geese. Types B and E, which often cause digestive tract diseases in ruminants, were both detected in pigs. *C. perfringens* type G, which produces the NetB toxin primarily causing necrotic enteritis in chickens, was found not only in chickens, but also in cows and geese (Table S1).

A comprehensive virulence gene analysis was performed on the genomes of the *C. perfringens* isolates, detecting 17 toxin genes including major toxins, adhesins, and hydrolases (Fig. 2). All tested isolates carried the alpha-toxin gene (*plc*), consistent with its role as a core virulence factor for this species. A total of 90 isolates carried the *cloSI*, *colA*, and *nagI* genes (90/91, 98.90%), while 89 isolates carried the *nagH* and *nanH* genes (89/91, 97.80%). Notably, the *cpb2* gene (55/91, 60.44%), encoding CPB2 toxin, which can cause animal enteritis/enterotoxemia and human digestive diseases, was detected across different animal species.

Toxin genes were abundant in *C. perfringens* from different food-producing animals. The majority of *C. perfringens* isolates (65/91, 71.43%) carried 10 or more virulence genes (Fig. 2). Although no strain carried all 17 tested toxin-related genes, the cow-derived isolate HLBR2 (toxin type B, sequence type ST1133, isolated from a bovine lung) carried 15 of them, including the classical toxin genes *cpb* and *etx* (Supplementary data 1).

### 3.2 *C. perfringens* isolates exhibit widespread antimicrobial resistance

**Fig. 3:**
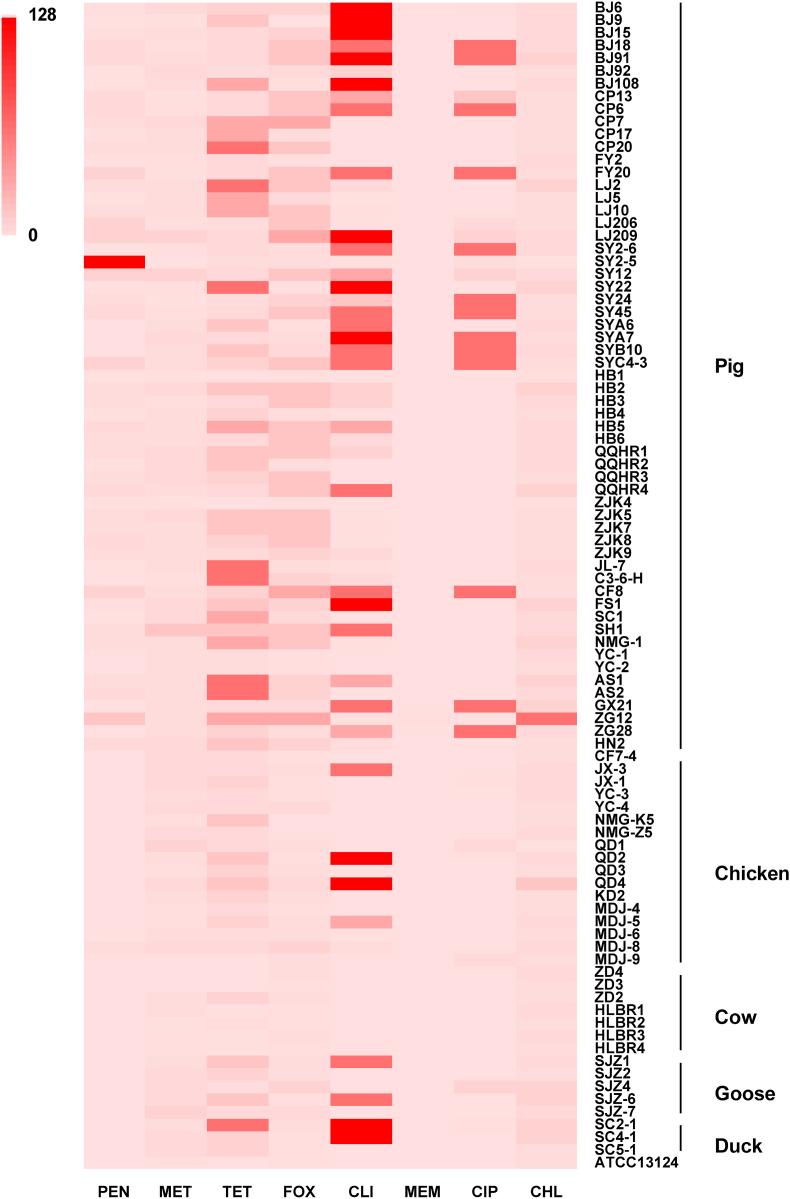
Multidrug-resistant heat maps of the *C. perfringens* isolates. Antibiotics abbreviations: penicillin (PEN), clindamycin (CLI), cefoxitin (FOX), chloramphenicol (CHL), meropenem (MEM), ciprofloxacin (CIP), tetracycline (TET), and metronidazole (MET).

**Fig. 4:**
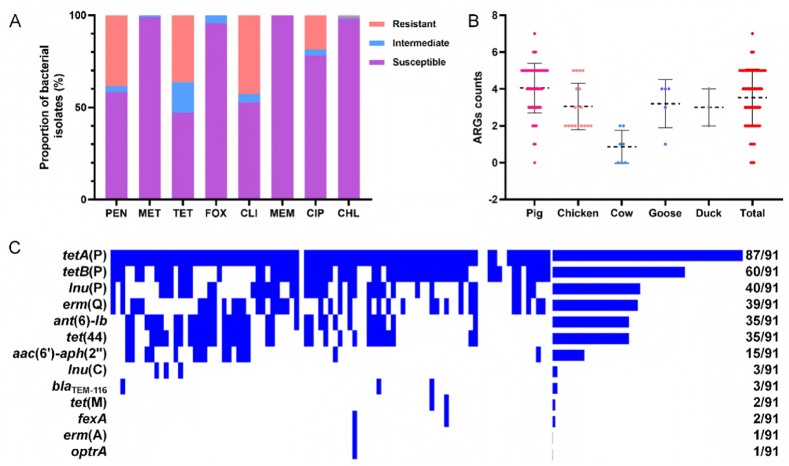
Antimicrobial Resistance in *C*. *perfringens*. (A) Antimicrobial susceptibility of the *C. perfringens* isolates. The MIC breakpoints were based on the Clinical and Laboratory Standards Institute (CLSI, 2018; CLSI, 2020) edition, with ciprofloxacin referenced to moxifloxacin. (B) Numbers of ARGs in the *C. perfringens* isolates. (C) Antimicrobial resistance genes (ARGs) of the *C. perfringens* isolates.

The susceptibility of the 91 clinical *C. perfringens* isolates to 8 antibiotics was tested (Fig. 3, Fig. 4, Table S2). Notably, 17.78% of the isolates (16/91) exhibited multidrug resistance phenotype, i.e., resistance to at least one antimicrobial agent of three different classes. The highest resistance rate was observed for clindamycin (39/91, 42.86%), followed by penicillin (35/91, 38.46%) and tetracycline (33/91, 36.26%). In addition, 18.68% (17/91) of isolates were resistant to ciprofloxacin. The most common resistance profile was resistance to penicillin-clindamycin-tetracycline, a pattern particularly prominent among porcine isolates (e.g., BJ91 and GX21).

The distribution of resistance varied among isolates from different animals, with porcine isolates presenting the most expanded resistance situation (Fig. 3, Fig. 4). Porcine isolates not only had the highest resistance rates to clindamycin and tetracycline, but the vast majority (>90%) of ciprofloxacin-resistant isolates also originated from pigs. In contrast, isolates from cows and poultry (chickens, ducks, and geese) had relatively narrow resistance spectra and remained susceptible to most tested antibiotics (Fig. 3, Fig. 4).

**Fig. 5:**
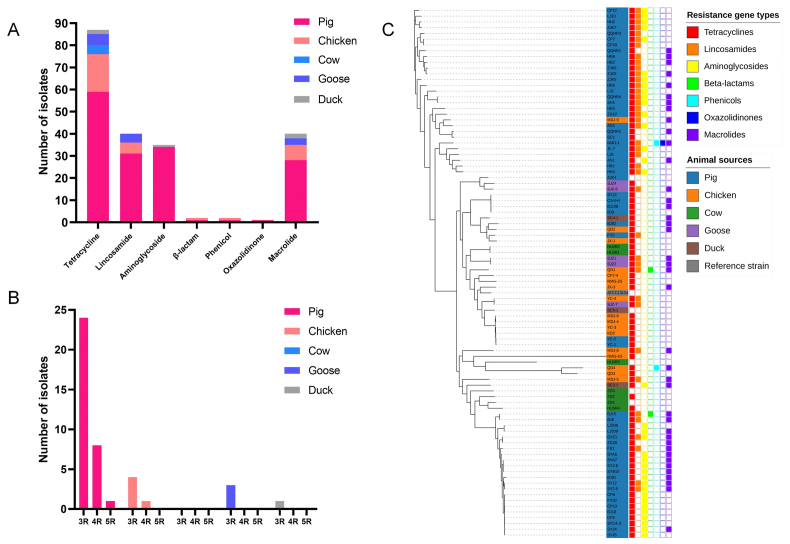
Resistance gene types in the *C. perfringens* isolates.

Based on WGS results, a comprehensive analysis of resistance genes was performed on the 91 *C. perfringens* isolates. A total of 14 genes was identified conferring resistance to tetracyclines, macrolides, lincosamides, aminoglycosides, β-lactams, phenicols, and oxazolidinones (Fig. 4, Fig. 5). Among them, tetracycline resistance genes were the most prevalent. The *tetA*(P) gene had an overwhelmingly high detection rate (94.51%, 86/91), and the *tetB*(P) gene was also common (65.93%, 60/91). The distribution of *tetB*(P) showed distinct host-source differences: highest prevalence in chicken isolates (86.67%, 13/15), followed by porcine isolates (61.02%, 36/59), and lower in cows (42.86%, 3/7). The overall carriage rates for the lincosamide (*lnu*(P)) and macrolide (*erm*(Q)) resistance genes were 43.96% (40/91) and 42.86% (39/91), respectively. The aminoglycoside resistance genes *ant*(6)-*Ib* and *aac*(6′)-*aph*(2″) were mainly enriched in porcine isolates, with carriage rates of 57.63% (34/59) and 23.73% (14/59), and were rare in isolates from other animals tested (Fig. 5, Supplementary data 1).

We categorized the resistance genes carried by the isolates and found that nearly half of the isolates (46.15%, 42/91) simultaneously carried three or more dfferent of resistance genes (Fig. 5). Among these, goose-origin isolates had the highest proportion (3/5, 60%), followed by pig-origin isolates (55.93%, 33/59), while duck- and chicken-origin isolates accounted for 33.33% (1/3) and 29.41% (5/17), respectively. The isolates derived from cows carried fewer than three types of resistance genes (Fig. 5). Notably, pig-derived *C. perfringens* contained the richest variety and abundance of resistance genes, serving as the primary reservoir for *C. perfringens* resistance genes. Among them, isolate NMG-1 simultaneously harbored resistance genes covering five classes of antibiotics: tetracyclines (*tetA*(P), *tetB*(P), *tet*(44)), macrolides (*erm*(A)), lincosamides (*lnu*(P)), phenicols (*fexA*), and oxazolidinones (*optrA*) (Fig. 5, Supplementary data 1).

### 3.3 Analysis of defense systems in *C. perfringens*

**Fig. 6:**
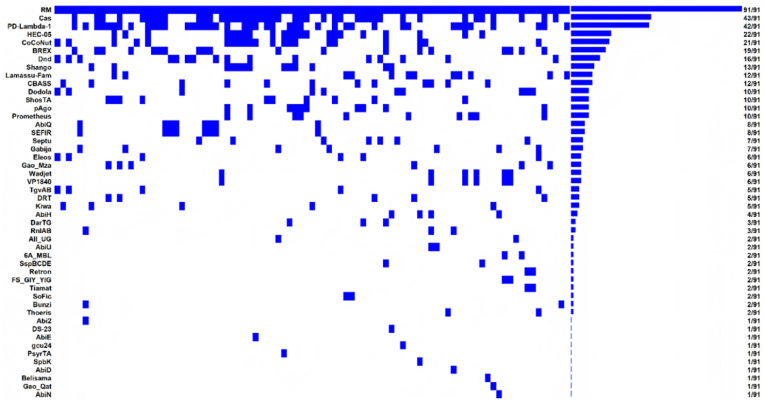
Defense systems of the *C. perfringens* isolates.

The distribution characteristics and diversification of bacterial defense systems are important for understanding bacterium-phage interactions and bacterial adaptive evolution. Therefore, through whole-genome analysis of defense systems, we systematically identified the distribution of 48 known bacterial defense systems among the 91 isolates, including Restriction-Modification (RM) systems, CRISPR-Cas systems, CBASS, Thoeris, Gabija, Abi family, and various other defense mechanisms (Fig. 6).

We found high variability in defense systems among different isolates (Fig. 6). Among the core defense systems, RM systems were universally present, detected in all 91 isolates (100%), highlighting their status as a fundamental defense mechanism. In contrast, the distribution of other defense systems was more limited, showing a clear strain-specific pattern. Among secondary defense systems, the CRISPR-Cas system (43/91, 47.25%) and PD-Lambda-1 system (42/91, 46.15%) were the most widely distributed specific defense systems. Systems like HEC-05, CoCoNut, and BREX were present in only about 20-30% of isolates, while the distribution of systems like Dnd, Shango, Lamassu-Fam, and CBASS was even more limited (below 20%). Notably, the number of defense systems carried by isolates also varied significantly (Fig. 5, S1). Some isolates, such as HB1-6 (all from pig anal swab samples in Shijiazhuang City, Hebei Province), carried a relatively rich array of defense systems, including RM, Cas, PD-Lambda-1, HEC-05, CoCoNut, Shango, among others. In contrast, some isolates, such as BJ9 and BJ15 (from pig anal swabs in Jinzhou City, Liaoning Province) carried only a few defense systems (Fig. 5, Supplementary data1).

Comparing the distribution of defense systems among isolates from different animals, we observed distinct host-specific distribution patterns. Porcine isolates exhibited the most diverse defense systems (Fig. 5, S1). Notably, certain defense systems showed clear host preferences. For example, the CoCoNut system was more common in porcine and bovine isolates, but less frequently detected in avian isolates. Conversely, the pAgo system was present in some avian isolates (e.g., CF7-4), but less common in porcine isolates (Supplementary data1).

### 3.4 STs and minimum spanning tree analysis

Through MLST analysis, the 91 isolates were classified into 59 distinct STs. Notably, 42 of these STs were newly discovered. We also submitted 37 new alleles and 42 new STs to the PubMLST database. Among the isolates, 17 contained new allele combinations resulting in 16 new STs. Furthermore, 37 isolates contained one or more new alleles, collectively forming 26 new STs (Table S3).

**Fig. 7:**
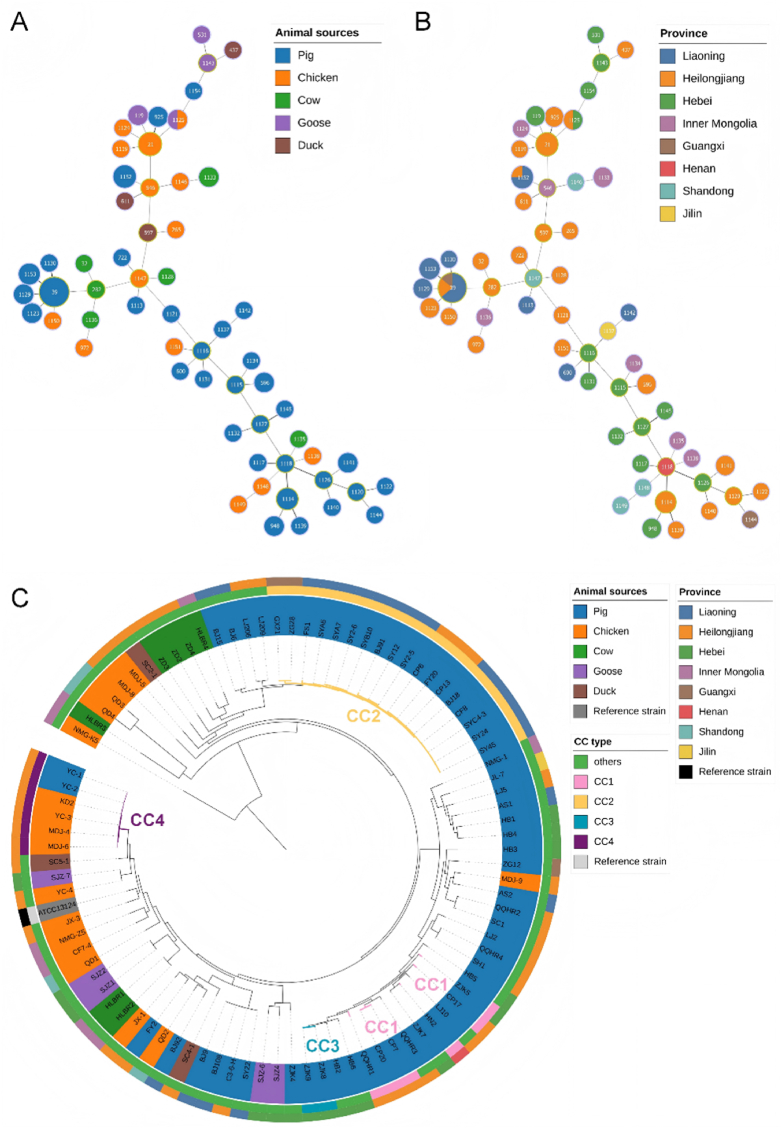
Phylogenetic analysis and population structure visualization of *C. perfringens* isolates across diverse animal sources. (A) The minimum spanning tree of the *C. perfringens* isolates from different animal source. (B) The minimum spanning tree of the *C. perfringens* isolates from different province. (C) Phylogenetic trees of the *C. perfringens* isolates from different sources.

Among all identified STs, ST39 (14/91, 15.38%) was the most prevalent overall, and all ST39 isolates originated from pigs, covering three provinces and six regions, accounting for about one-quarter of pig-origin isolates (14/59, 23.73%). The second most prevalent ST, ST21 (4/91, 4.40%), consisted entirely of isolates from chicken samples in three different regions of Heilongjiang Province, accounting for about one-quarter of chicken-origin isolates (4/17, 23.53%) (Fig. 7). ST1114, ST1152, and ST1153, each containing three isolates (3/91, 3.30%), all originated from pigs. ST119, ST596, ST925, ST948, ST1123, ST1125, ST1129, and ST1133 each contained two isolates (2/91, 2.20%). The remaining 46 STs each were represented by a single strain (1/91, 1.10%) (Fig. 7).

STs of *C. perfringens* differed significantly among animals and source regions (Fig. 7). The 59 pig-origin *C. perfringens* isolates from seven provinces comprised 33 STs, with ST39 (14/59, 23.73%) being the most prevalent ST, covering Heilongjiang, Liaoning, and Guangxi provinces, followed by ST1114, ST1152, and ST1153, all from Heilongjiang Province (3/59, 5.08%). Among the 17 chicken-origin isolates, ST21 (4/17, 23.53%) was the major prevalent ST, originating from three different regions in Heilongjiang Province; the remaining 13 isolates all belonged to different STs. The seven dairy cow-origin isolates comprised six STs, with ST1133 containing two isolates (2/7, 28.57%). The five goose-origin isolates comprised four STs, with ST119 containing two isolates (2/5, 40.00%). The three duck-origin isolates comprised three distinct STs (Fig. 7).

### 3.5 Phylogenetic and genetic diversity analysis

A clonal complex (CC) was defined as a group of isolates sharing seven or more identical alleles across the housekeeping genes. The 91 isolates mainly comprised four CCs (CC1-CC4), accounting for 34.07% (31/91) of all isolates (Fig. 7). CC1 contained six of the 91 isolates (6.59%) and had the most STs, namely ST1114, ST1139, ST1126, and ST1118. These STs were from three provinces (Heilongjiang, Henan, and Hebei) and all obtained from pig samples. CC2, which included the most isolates, comprised ST39, ST1130, and ST1153, accounting for 19.78% of all isolates. These isolates originated from pig farms in Heilongjiang, Liaoning, and Guangxi provinces (Fig. 7). CC3 contained only ST1145 and ST1127, with two isolates from a pig farm in Zhangjiakou City, Hebei Province. CC4 contained ST21 and ST925, with all six isolates originating from chicken farms in 3 different cities in Heilongjiang Province.

The results of the phylogenetic tree and the minimum spanning tree were largely consistent but not entirely congruent. Isolates of the same CC typically clustered together. Isolates from CC2, CC3, and CC4 all formed distinct clusters in the phylogenetic tree, but exceptions existed. For example, the six isolates in CC1 were assigned to different branches in the phylogenetic tree (Fig. 7).

### 3.6 Gene profiles of the dominant ST39 isolates

**Fig. 8:**
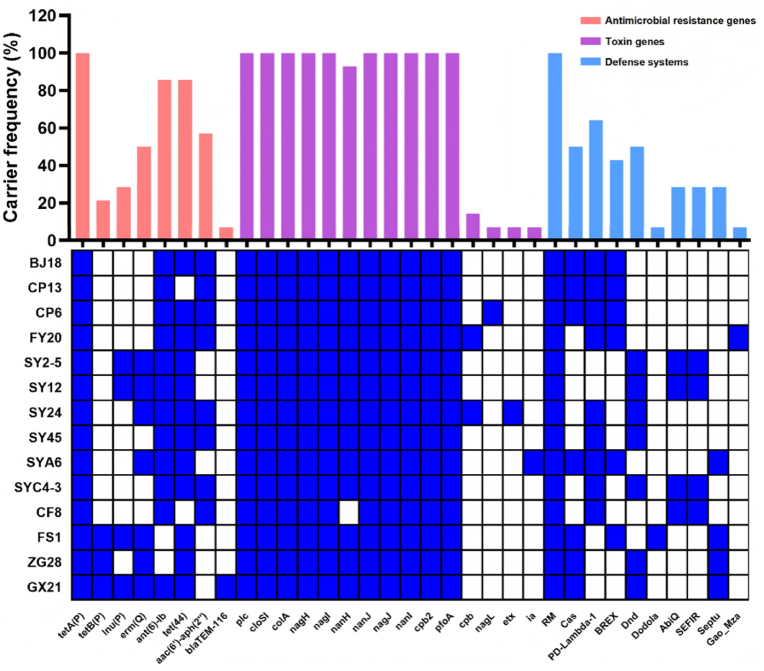
Genetic profile of *C. perfringens* ST39. The heatmap shows the carriage status of various genetic elements in the genome of *C. perfringens* ST39, including antimicrobial resistance genes, virulence factors, and defense systems. Each bar represents the carriage rate of a specific gene.

Our analysis of the gene profiles of the dominant ST39 isolates revealed that all isolates collectively harbored eight types of resistance genes, and 64.29% (9/14) of the isolates exhibited a multidrug resistance (MDR) phenotype (Fig. 8). Tetracycline resistance genes were the most prevalent, including *tetA*(P) (14/14, 100%), *tet*(44) (12/14, 85.71%), and *tetB*(P) (3/14, 21.43%). This was followed by aminoglycoside resistance genes (*ant*(6)-*Ib* (12/14, 85.71%) and *aac*(6′)-*aph*(2″) (8/14, 57.14%)) and macrolide resistance gene (*erm*(Q) (7/14, 50%)). Lincosamide resistance gene (*lnu*(P) (4/14, 28.57%)) and β-lactam resistance gene (*bla*_TEM-116_ (1/14, 7.14%)) were the least common (Fig. 8). All isolates contained ten or more virulence genes, and every isolate carried virulence factors involved in mucosal colonization or nutrient acquisition (*colA*, *nagH*, *nagI*, *nanH*, *nanJ*, *nagJ*, *nanI*, *nagK*, and *nagL*) as well as the pore-forming toxin gene *pfoA* (Fig. 8). In terms of toxin typing, type A was predominant (11/14 isolates), with one isolate each belonging to types B, C, and E (Supplementary data 1). The ST39 isolates collectively possessed ten defense systems. The defense system profile showed that RM, PD-Lambda-1, Cas, and Dnd (>50% prevalence) were universally present. These BREX, AbiQ, SEFIR, and Septu were high-frequency systems (20%-50%), while Dodola and Gao_Mza were rare (<10%) (Fig. 8). These findings indicated that ST39 represented a widely disseminated bacterial clonal group in swine populations, characterized by multidrug resistance, strong intestinal colonization capacity, and a diverse arsenal of defense systems.

## 4 Discussion

As a core component of the dietary structure, meat (pork, beef, and chicken) is not only a crucial nutritional source for humans but also a major vehicle for pathogen transmission. *C. perfringens* is a significant pathogen causing foodborne illnesses. Its ability to produce spores allows it to persist on food surfaces (Savard et al., 2026), making it difficult to eliminate, and it is associated with human food poisoning, animal necrotic enteritis, antibiotic-associated diarrhea (AAD), and other diseases (Kiu and Hall, 2018). In this study, we focused on *C. perfringens* of food-producing animal origin, which may pose a threat to human health, and analyzed the genomic characteristics of isolates from different regions and animal sources. Consistent with previously reported data, *C. perfringens* exhibits genetic diversity and carries a highly enriched repertoire of toxin genes, antibiotic resistance genes, and defense systems (Abdel-Glil et al., 2021; Wu et al., 2025; Zhong et al., 2023a).

The isolation rate of *C*. *perfringens* varies greatly across different countries, regions, and animal sample sources. Most reported studies in China have focused on single animal species within limited geographic areas, with isolation rates ranging from 14.7% to 72% (Li et al., 2021; Liu et al., 2025; Wu et al., 2022; Xiu et al., 2020). The majority of the samples collected in this study originated from Northeast China—a key livestock-producing region—and confirmed an overall carriage rate of 26.07%. This value falls within the range of previously reported rates and is comparable to the 22.2% isolation rate reported in cattle, pig, and poultry slaughterhouses in France (Tran et al., 2026), as well as the 25.37% carriage rate reported in poultry farms in Pakistan (Haider et al., 2022). Notably, the isolation rates in intestinal content and feces were significantly higher than those in anal swabs and organ samples, which aligns with the fact that *C. perfringens* colonizes the intestinal tract; positivity in organ samples may indicate true infection or post-mortem translocation.

The pathogenicity of *C. perfringens* is determined by a diverse array of toxins and other virulence factors, with toxins exhibiting specific host tropisms. Different toxin types can cause clinically distinct diseases in different hosts (Camargo et al., 2024). Among the *C. perfringens* isolates obtained in this study, all toxin types were identified except for type F (primarily causing human food poisoning) and type D (causing enterotoxemia in ruminants) (Table S1). The majority were type A (52.75%), which is the predominant type globally and is associated with human food poisoning and gas gangrene in livestock. *C. perfringens* type C, which can cause diarrhea in piglets, was present in nearly half of the porcine samples. Notably, *C. perfringens* of different toxin types were also identified in non-typical hosts. For instance, type G isolates, which produce NetB toxin and primarily cause necrotic enteritis in chickens, were also detected in dairy cow-derived isolates. Similarly, type B isolates, which carry the ETX toxin and mainly cause enterotoxemia in ruminants, were found among porcine-derived isolates. This phenomenon is not an isolated case. A survey conducted in French slaughterhouses in 2026 also found that type D toxin, typically prevalent in ruminants, was detected in bovine isolates (Tran et al., 2026). This may be related to the transfer of toxin gene-carrying plasmids between different hosts via conjugation or other mechanisms (Sayeed et al., 2007; Li et al., 2013).

We detected a total of 17 toxin-related genes, with 71.43% of isolates carrying ten or more virulence genes (Fig. 2). The maximum number of virulence genes carried by a single strain was 15 out of the 17 genes in the virulence profile. This indicates that food producing animal-derived *C. perfringens* isolates possess a broad spectrum of virulence genes, posing a potential threat to food safety. Genes such as *cloSI*, *colA*, and *nagI* (98.90%), as well as *nagH* and *nanH* (97.80%), were detected in almost all isolates. Genes like *nanJ* (75.82%), *nagJ* (74.73%), *nanI* (69.23%), *cpb2* (60.44%), and *pfoA* (60.44%) also had high detection rates. The detection rates for *cpb*, *etx*, *ia*, and *netB* were low (1.10%-41.76%). The detection rate for *nagL* (31.89%) was lower than reported in other literature (Wu et al., 2025). The analysis of the virulence gene profile in this study revealed that the pathogenicity of *C. perfringens* relies on a multi-layered, synergistic network of virulence factors, a notion consistent with recent genomic studies demonstrating that this pathogen's broad and heterogeneous toxin arsenal underpins its ability to cause diverse diseases across multiple host species (Camargo et al., 2024; Geier et al., 2021). Nearly all isolates are equipped with genes for disrupting tissue barriers (*cloSI* and *colA*) and an array of sialidases for degrading mucus and promoting colonization (*nagH*, *nagI*, *nanH*, *nanJ*, *nagJ* and *nanI*). The *colA* gene, encoding kappa-toxin (collagenase), is known to be positively regulated by the VirR/VirS two-component signal transduction system, which coordinately regulates the expression of multiple extracellular toxin genes in *C. perfringens* (Ba-Thein et al., 1996). Furthermore, most isolates also possess effector toxins for direct cell lysis (*pfoA*) and inducing intestinal damage (*cpb2*). The pore-forming toxin perfringolysin O (PFO, encoded by *pfoA*) is also a member of the VirR/VirS regulon (Ba-Thein et al., 1996), while CPB2 toxin has been shown to induce oxidative damage, disrupt tight junctions, and trigger inflammatory responses and apoptosis in porcine small intestinal epithelial cells (Luo et al., 2020). Notably, *C. perfringens* strains can produce up to three distinct sialidases (NanH, *Nan*I, and NanJ), each with different enzymatic properties, optimal temperatures, and sensitivities to inhibitors (Li and McClane Bruce, 2014). These sialidases not only play nutritional roles by releasing sialic acids from host glycoconjugates but also function as virulence factors that facilitate adherence to host cells and enhance toxin binding (Therit et al., 2015). The genetic diversity and functional redundancy among sialidases may allow *C. perfringens* to adapt to diverse host environments. Genes like *cpb*, *etx*, *ia*, and *netB* are classical toxins, and their presence often determines a strain's ability to cause specific severe diseases (e.g. necrotic enteritis or enterotoxemia). Many of these toxin genes, including *netB*, are located on conjugative plasmids that can be transferred horizontally between different *C. perfringens* strains (Lacey Jake et al., 2017). This explains why the same bacterial species can cause diseases with vastly different clinical manifestations. Notably, the detection rate of the sialidase gene *nagL* (31.89%) was significantly lower than in other studies (Wu et al., 2025), which may reflect the unique genetic background of prevalent isolates from food-producing animals in China. Its absence might be compensated for by other functionally redundant sialidases with overlapping but distinct substrate specificities and properties (Li et al., 2013; Therit et al., 2015). The different combinations of these core and auxiliary virulence factors collectively determine a strain's host preference and disease severity.

The cow-derived isolate HLBR2, which carries 15 out of the 17 toxin-related genes detected in this study, deserves special attention. HLBR2 is a toxin type B strain co-harboring *cpb* (beta toxin) and *etx* (epsilon toxin). The coexistence of these two classical toxins in a single bovine isolate is unusual, as *cpb* is typically associated with severe enteritis in neonatal animals while *etx* is a hallmark of enterotoxemia in ruminants. Although HLBR2 belongs to ST1133, which is not a major epidemic clone, such a high toxin gene load may confer pathogenic potential under immunocompromised or stress conditions. Furthermore, HLBR2 carries a diverse array of defense systems, including RM, BREX, Wadjet, VP1840, and FS_GIY_YIG, which may protect the strain from foreign mobile genetic elements and contribute to the stability of its exceptionally high toxin gene repertoire (Appendix A. Supplementary data 1). The results obtained from this isolate underscore the need for continuous genomic surveillance of *C. perfringens* in food-producing animals, as cows may serve as reservoirs for hypervirulent strains carrying unconventional combinations of classical toxin genes.

AMR is a global public health concern. Therefore, we investigated the antimicrobial susceptibility of *C. perfringens* isolates. The results showed high resistance rates to clindamycin (42.86%), penicillin (38.46%), and tetracycline (36.26%) (Fig. 3, Fig. 4). This resistance pattern (penicillin-clindamycin-tetracycline) was observed in our study. These resistance rates are comparable to those reported in a recent genomic study of food animal-derived *C. perfringens* in China, which found clindamycin resistance in 39.5% of isolates and tetracycline resistance in 32.6% (Wu et al., 2025). This result suggests that these three types of antimirobial agents should be avoided in the treatment of *C*. *perfringens* diseases. The main ARGs identified in *C. perfringens* isolates were *tetA*(P), *tetB*(P), *lnu*(P), *erm*(Q), and *ant*(6)-*Ib*. A total of 46.15% of isolates carried three or more types of resistance genes (Fig. 5). The high prevalence of tetracycline resistance genes is consistent with previous findings that *tetA*(P) and *tetB*(P) are the most frequently identified resistance genes in *C. perfringens*, present in 93.0% and 79.0% of isolates, respectively (Wu et al., 2025). The prevalence of MDR phenotype may be related to the co-location of these ARGs on a single mobile genetic element (Revitt-Mills et al., & Rood, 2021). It is noteworthy that there is not a complete correlation between the phenotype and genotype of resistance genes. Only three strains carrying the *bla*_TEM-116_ gene exhibited a penicillin resistance rate of 38.46%. This discrepancy may be attributed to the presence of altered penicillin-binding proteins (PBPs) or other undetected resistance mechanisms. Furthermore, a large number of strains carrying tetracycline resistance genes did not show a corresponding resistant phenotype. We speculate that this inconsistency arises because CLSI has not established specific MIC breakpoints for *C*. *perfringens*, and the application of breakpoints intended for general anaerobic bacteria may not be accurate for this species. The resistance profiles varied significantly depending on the host source. Porcine-derived isolates ranked first in terms of the diversity and abundance of ARGs, as well as proportion of multidrug resistance. This is attributable to the historical and current use of antibiotics (especially tetracyclines, lincosamides, and β-lactams for therapy or growth promotion) in intensive pig farming, creating a powerful environmental selection pressure.

Oxazolidinones are considered a last line of defense against infections caused by Gram-positive bacteria. The *optrA* resistance gene which mediates co-resistance to oxazolidinones (e.g., linezolid and tedizolid) and phenicols (e.g., florfenicol and chloramphenicol), was first identified in *Enterococcus faecalis* and *Enterococcus faecium* of human and animal origin (Wang et al., 2015). In our study, strain NMG-1 was found to carry the *optrA* resistance gene, which has also been reported in plasmids of *C. perfringens* in both China and South Korea (Kim et al., 2025; Zhou et al., 2020). Notably, a recent genomic study identified an *optrA*-positive *C. perfringens* strain QHY-2 from Tibetan sheep in Qinghai province, and the *optrA* plasmid pQHY-2 was found to belong to a novel plasmid type distinct from the pCW3-like and pCP13-like plasmids that typically carry toxin genes. This novel plasmid type can also harbor multiple other ARGs and may coexist with toxin-encoding plasmids, posing a potential threat to public health (Wu et al., 2023). There is a risk that this gene could be transmitted from animals to humans through the food chain. From a “One Health” perspective, as a transferable multidrug resistance gene, the prevalence and dissemination of isolates carrying *optrA* may pose a significant threat to public health.

With the long-term and often inappropriate use of antibiotics, the resistance of *C. perfringens* has gradually increased, and the incidence of diseases caused by this bacterium has risen, causing significant economic losses to the farming industry and impacting animal husbandry development and public health safety (Yan et al., 2024; Zhong et al., 2023). The World Health Organization (WHO) has listed AMR as a major global public health threat. Bacteriophages (phages), as “obligate predators” of bacteria, have become a popular subject for research into novel biological agents for treating bacterial diseases (Rafiq et al., 2025; Zhang et al., 2024). In the environmental “arms race” between phages and bacteria, phages can accelerate bacterial evolution through horizontal gene transfer, while bacteria employ defense systems to maintain genomic stability, and phages, in turn, develop anti-defense mechanisms (Hampton et al., 2020). Studies have found that bacterial resistance to phages may alter their metabolism or virulence, indirectly affecting antibiotic susceptibility (Tu et al., 2025). Understanding the defense system profile of *C. perfringens* and dynamically monitoring potential therapeutic escape resulting from co-evolution is of significant importance for developing phage therapies against such highly resistant pathogens. Currently, comprehensive genomic studies on the diversity and evolution of phage defense systems in food producing animal-derived *C. perfringens* are still lacking. Notably, a previous genomic analysis revealed the absence of CRISPR defense systems in >70% of *C. perfringens* strains examined, highlighting the variability and strain-specific nature of defense mechanisms in this species (Kiu et al., 2017). This study is - to the best of our knowledge - the first to systematically depict the panorama of defense systems in food producing animal-derived *C. perfringens*. The universal presence of the RM system in all isolates establishes its indispensable role as a core, fundamental defense. However, the highly strain-specific and host-preferential distribution of other defense systems (e.g., CRISPR-Cas, PD-Lambda-1, etc.) reveals more complex evolutionary dynamics. Porcine-derived isolates exhibited the richest diversity of defense systems, which may be directly related to the intense phage and gene flow pressure exerted by their high-density, high-mobility farming environment. The host preference of specific systems (e.g., CoCoNut and pAgo) suggests adaptive customization of defense mechanisms through long-term interaction with specific intestinal ecologies. Most importantly, the distribution pattern of defense systems may be closely related to a strain's genomic plasticity and adaptive evolutionary potential. We hypothesize that isolates with a paucity of defense systems might be more prone to acquiring foreign resistance and virulence genes via horizontal gene transfer, enabling rapid evolution. Conversely, isolates with a rich arsenal of defense systems likely possess greater genomic stability in complex microbial competitions.

MLST and phylogenetic analyses in this study revealed extremely high genetic diversity in the *C. perfringens* of food-producing animal origin population in China, with over 70% of STs being newly identified. This is consistent with previous MLST studies of *C. perfringens* in China, which reported 135 STs among 186 isolates, including 93 new STs (Zhong et al., 2023b), as well as a study on duck production chains that identified 47 STs from 65 representative isolates (Xu et al., 2021). The high proportion of novel STs suggests active local evolution and genetic recombination. Against this background, ST39 (porcine-derived) and ST21 (poultry-derived) emerged as clearly dominant clones. They have achieved cross-geographic dissemination within pig and chicken populations, respectively, indicating they have acquired significant adaptive advantages, thereby maintaining their high-fitness phenotype. Clonal complex (CC) analysis further supports the recent clonal expansion of ST39 (CC2). However, the dispersal of CC1 isolates in the phylogenetic tree also suggests that MLST sometimes does not fully align with whole-genome evolutionary history, which may be caused by horizontal transfer of accessory genomes. Previous studies have also noted that within the same ST complex, genetically unrelated relationships or potential clustering/transmission events can be further recognized by cgMLST and cgSNP, illustrating that these higher-resolution methods are valuable in defining outbreaks and transmission events (Zhong et al., 2023a).

ST39 *C. perfringens* has been reported in recent years in piglets from Jiangxi Province and in ducks and the environment from Shandong Province, China (Xiu et al., 2020; Xu et al., 2021). Furthermore, the ST39 *C. perfringens* strain in the PubMLST database is the NobL1 strain submitted from the USA in 1996. We posit that this occurrence of ST39 as a dominant strain across national borders is not coincidental. ST39 isolates consistently carry a set of virulence factors associated with mucosal colonization and nutrient acquisition (e.g., *colA*, *nagH*, *nanH*, etc.). These factors act like “anchors” and “scissors,” helping the bacteria adhere firmly to the host intestinal epithelium and degrade the mucus layer to obtain carbon sources. This greatly facilitates long-term colonization, proliferation, and continuous shedding via feces. All isolates are multidrug-resistant, harboring a rich repertoire of resistance genes to withstand antibiotic treatment pressure in intensive farming. Moreover, their abundant defense systems may, on one hand, make them more difficult to eliminate via phage therapy and, on the other hand, potentially stabilize their existing resistance and virulence genotypes, protecting against phage infection and horizontal gene transfer of exogenous MGEs. Overall, the population structure shows clear host association. However, successful clones like ST39 warn us that efficient livestock industry circulation networks may enable specific high-risk lineages to overcome geographical constraints, constituting a widespread food safety risk. Future monitoring should focus on the dynamics of such dominant clones and the co-evolutionary trends of their virulence and resistance.

## 5 Conclusion

This study systematically revealed the genetic characteristics of *C. perfringens* from food-producing animals in China through whole-genome sequencing. The isolates harbor abundant toxin and antimicrobial resistance genes, exhibiting high genetic diversity, with the multidrug-resistant ST39 clone being particularly prominent. This clone integrates strong colonization capacity, MDR, and a diverse arsenal of phage defense systems, demonstrating high adaptability and posing a significant risk to food safety and public health. The findings underscore the necessity for enhanced monitoring of antimicrobial resistance and the implementation of antibiotic reduction strategies at the farm level to curb the spread of such high-risk clones.

## Funding

This work was supported by Prevention and Control of Emerging and Major Infectious Diseases-National Science and Technology Major Project (2025ZD01900100), Heilongjiang Provincial Natural Science Foundation of China (LH2024C061), Central Public-interest Scientific Institution Basal Research Fund (1610302024001) and National Key Research and Development Program of China (2024YFC2607402).

## Declaration of competing interest

The authors declare that they have no known competing financial interests or personal relationships that could have appeared to influence the work reported in this paper.
