# Supplementary material for: Toxin gene profiles, antibiotic resistance, and genetic diversity of Clostridium perfringens from food-producing animals: a whole-genome sequencing study with implications for food safety
Source: Curr Res Food Sci. 2026 Jun 1;12:101460. doi: 10.1016/j.crfs.2026.101460 (PMC13251782; doi:10.1016/j.crfs.2026.101460)
Supplement: Multimedia component 2 [file mmc2.docx]

**Table S1.** Toxin types of the *C. perfringens* from different animal sources.

| Animal sources | Toxin types | | | | |
| --- | --- | --- | --- | --- | --- |
|  | A | B | C | E | G |
| Pig | 29 | 2 | 27 | 1 | 0 |
| Chicken | 11 | 0 | 4 | 0 | 2 |
| Cow | 3 | 1 | 2 | 0 | 1 |
| Goose | 2 | 0 | 2 | 0 | 1 |
| Duck | 3 | 0 | 0 | 0 | 0 |
| Total | 48 | 3 | 35 | 1 | 4 |

**Table S2.** Minimal inhibitory concentrations of the 91 *C. perfringens* isolates.

| **Antimicrobial Agent(s)** | **Number of isolates for which the MIC value (mg/L) is** | | | | | | | | | | | |
| --- | --- | --- | --- | --- | --- | --- | --- | --- | --- | --- | --- | --- |
|  | **0.125** | **0.25** | **0.5** | **1** | **2** | **4** | **8** | **16** | **32** | **64** | **128** |  |
| Penicillin | 34 | 10 | 9 | 3 | 17 | 10 | 6 | 1 | 0 | 0 | 1 |  |
| Cefoxitin | 0 | 0 | 3 | 20 | 21 | 8 | 11 | 24 | 4 | 0 | 0 |  |
| Chloramphenicol | 0 | 1 | 1 | 2 | 38 | 34 | 13 | 1 | 0 | 1 | 0 |  |
| Clindamycin | 23 | 15 | 2 | 6 | 2 | 4 | 4 | 1 | 6 | 15 | 13 |  |
| Ciprofloxacin | 4 | 46 | 17 | 3 | 1 | 3 | 3 | 1 | 0 | 13 | 0 |  |
| Tetracycline | 7 | 0 | 1 | 0 | 9 | 26 | 15 | 16 | 9 | 8 | 0 |  |
| Meropenem | 76 | 1 | 14 | 0 | 0 | 0 | 0 | 0 | 0 | 0 | 0 |  |
| Metronidazole | 0 | 6 | 5 | 15 | 36 | 24 | 4 | 1 | 0 | 0 | 0 |  |


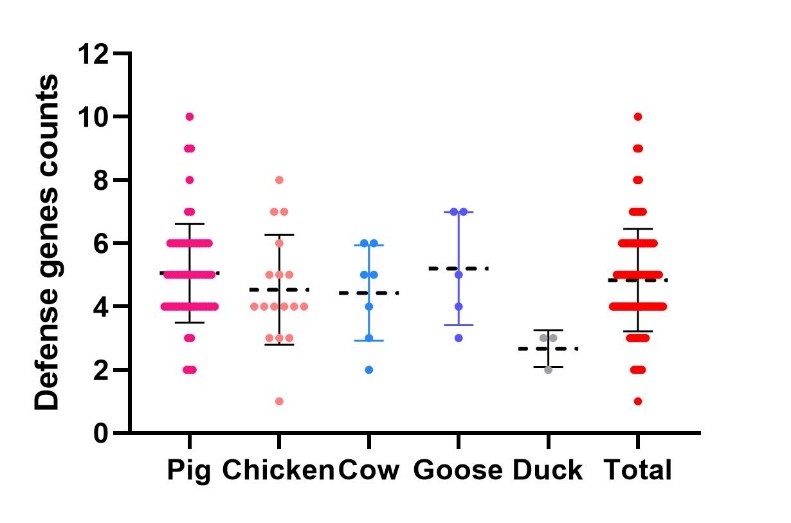


**Fig. S1** Numbers of defense genes in the *C. perfringens* isolates.

**Table S3.** Allelic profile of *C. perfringens* for MLST

| **Isolate** | **ST** | ***colA*** | ***groEL*** | ***sodA*** | ***plc*** | ***gyrB*** | ***sigK*** | ***pgk*** | ***nadA*** |
| --- | --- | --- | --- | --- | --- | --- | --- | --- | --- |
| BJ6 | 1129 | 33 | 4 | 228 | 39 | 6 | 4 | 3 | 19 |
| BJ9 | 1152 | 4 | 29 | 19 | 43 | 1 | 173 | 4 | 240 |
| BJ15 | 1129 | 33 | 4 | 228 | 39 | 6 | 4 | 3 | 19 |
| BJ18 | 39 | 33 | 4 | 40 | 39 | 7 | 4 | 3 | 19 |
| BJ91 | 1130 | 33 | 4 | 229 | 39 | 7 | 4 | 3 | 19 |
| BJ92 | 1113 | 19 | 5 | 3 | 5 | 5 | 122 | 3 | 1 |
| BJ108 | 1152 | 4 | 29 | 19 | 43 | 1 | 173 | 4 | 240 |
| CP13 | 39 | 33 | 4 | 40 | 39 | 7 | 4 | 3 | 19 |
| CP6 | 39 | 33 | 4 | 40 | 39 | 7 | 4 | 3 | 19 |
| CP7 | 1114 | 11 | 81 | 141 | 126 | 52 | 8 | 6 | 44 |
| CP17 | 1114 | 11 | 81 | 141 | 126 | 52 | 8 | 6 | 44 |
| CP20 | 1114 | 11 | 81 | 141 | 126 | 52 | 8 | 6 | 44 |
| FY2 | 722 | 19 | 5 | 3 | 5 | 1 | 2 | 3 | 105 |
| FY20 | 39 | 33 | 4 | 40 | 39 | 7 | 4 | 3 | 19 |
| LJ2 | 1120 | 9 | 81 | 7 | 12 | 52 | 10 | 6 | 44 |
| LJ5 | 1121 | 11 | 10 | 6 | 11 | 1 | 9 | 2 | 1 |
| LJ10 | 1122 | 11 | 81 | 6 | 12 | 52 | 10 | 6 | 44 |
| LJ206 | 1123 | 33 | 4 | 40 | 31 | 113 | 19 | 3 | 19 |
| LJ209 | 1123 | 33 | 4 | 40 | 31 | 113 | 19 | 3 | 19 |
| SY2-6 | 1153 | 33 | 4 | 40 | 285 | 7 | 4 | 3 | 19 |
| SY2-5 | 39 | 33 | 4 | 40 | 39 | 7 | 4 | 3 | 19 |
| SY12 | 39 | 33 | 4 | 40 | 39 | 7 | 4 | 3 | 19 |
| SY22 | 1152 | 4 | 29 | 19 | 43 | 1 | 173 | 4 | 240 |
| SY24 | 39 | 33 | 4 | 40 | 39 | 7 | 4 | 3 | 19 |
| SY45 | 39 | 33 | 4 | 40 | 39 | 7 | 4 | 3 | 19 |
| SYA6 | 39 | 33 | 4 | 40 | 39 | 7 | 4 | 3 | 19 |
| SYA7 | 1153 | 33 | 4 | 40 | 285 | 7 | 4 | 3 | 19 |
| SYB10 | 1153 | 33 | 4 | 40 | 285 | 7 | 4 | 3 | 19 |
| SYC4-3 | 39 | 33 | 4 | 40 | 39 | 7 | 4 | 3 | 19 |
| HB1 | 1131 | 249 | 10 | 6 | 278 | 2 | 9 | 6 | 10 |
| HB2 | 1132 | 250 | 177 | 6 | 126 | 52 | 8 | 6 | 44 |
| HB3 | 1115 | 9 | 144 | 6 | 12 | 4 | 9 | 6 | 44 |
| HB4 | 1116 | 13 | 10 | 6 | 12 | 2 | 9 | 6 | 10 |
| HB5 | 948 | 11 | 10 | 141 | 126 | 52 | 10 | 6 | 44 |
| HB6 | 1117 | 9 | 177 | 141 | 126 | 52 | 9 | 6 | 44 |
| QQHR1 | 1139 | 11 | 81 | 231 | 126 | 52 | 8 | 6 | 44 |
| QQHR2 | 596 | 11 | 41 | 6 | 12 | 4 | 9 | 6 | 44 |
| QQHR3 | 1140 | 254 | 81 | 231 | 126 | 52 | 10 | 6 | 44 |
| QQHR4 | 1141 | 255 | 63 | 141 | 139 | 52 | 10 | 6 | 44 |
| ZJK4 | 1154 | 64 | 4 | 46 | 176 | 2 | 176 | 22 | 240 |
| ZJK5 | 1126 | 9 | 81 | 141 | 126 | 52 | 10 | 6 | 44 |
| ZJK7 | 948 | 11 | 10 | 141 | 126 | 52 | 10 | 6 | 44 |
| ZJK8 | 1145 | 9 | 177 | 6 | 281 | 52 | 8 | 6 | 44 |
| ZJK9 | 1127 | 9 | 177 | 6 | 12 | 52 | 8 | 6 | 44 |
| JL-7 | 1137 | 13 | 10 | 6 | 12 | 2 | 8 | 2 | 234 |
| C3-6-H | 1152 | 4 | 29 | 19 | 43 | 1 | 173 | 4 | 240 |
| CF8 | 39 | 33 | 4 | 40 | 39 | 7 | 4 | 3 | 19 |
| FS1 | 39 | 33 | 4 | 40 | 39 | 7 | 4 | 3 | 19 |
| SC1 | 596 | 11 | 41 | 6 | 12 | 4 | 9 | 6 | 44 |
| SH1 | 1141 | 255 | 63 | 141 | 139 | 52 | 10 | 6 | 44 |
| NMG-1 | 1134 | 3 | 10 | 6 | 126 | 4 | 9 | 6 | 235 |
| YC-1 | 925 | 3 | 1 | 3 | 4 | 3 | 154 | 1 | 1 |
| YC-2 | 925 | 3 | 1 | 3 | 4 | 3 | 154 | 1 | 1 |
| AS1 | 1142 | 248 | 12 | 6 | 11 | 2 | 8 | 28 | 1 |
| AS2 | 600 | 11 | 128 | 7 | 12 | 2 | 9 | 6 | 1 |
| GX21 | 39 | 33 | 4 | 40 | 39 | 7 | 4 | 3 | 19 |
| ZG12 | 1144 | 13 | 209 | 7 | 12 | 52 | 9 | 6 | 44 |
| ZG28 | 39 | 33 | 4 | 40 | 39 | 7 | 4 | 3 | 19 |
| HN2 | 1118 | 9 | 81 | 141 | 126 | 52 | 8 | 6 | 44 |
| CF7-4 | 546 | 4 | 4 | 1 | 4 | 3 | 2 | 1 | 1 |
| JX-3 | 1119 | 194 | 1 | 3 | 88 | 3 | 147 | 1 | 1 |
| JX-1 | 265 | 19 | 60 | 1 | 5 | 5 | 2 | 2 | 3 |
| YC-3 | 21 | 3 | 1 | 3 | 4 | 3 | 2 | 1 | 1 |
| YC-4 | 1125 | 64 | 3 | 63 | 4 | 2 | 2 | 1 | 11 |
| NMG-K5 | 1138 | 253 | 186 | 198 | 280 | 100 | 175 | 105 | 236 |
| NMG-Z5 | 1124 | 6 | 19 | 3 | 10 | 3 | 5 | 20 | 1 |
| QD1 | 1146 | 4 | 63 | 232 | 1 | 3 | 2 | 1 | 1 |
| QD2 | 1147 | 19 | 5 | 1 | 5 | 1 | 177 | 3 | 1 |
| QD3 | 1148 | 62 | 15 | 119 | 282 | 73 | 73 | 84 | 237 |
| QD4 | 1149 | 257 | 139 | 119 | 149 | 73 | 91 | 65 | 122 |
| KD2 | 21 | 3 | 1 | 3 | 4 | 3 | 2 | 1 | 1 |
| MDJ-4 | 21 | 3 | 1 | 3 | 4 | 3 | 2 | 1 | 1 |
| MDJ-5 | 972 | 75 | 196 | 118 | 177 | 49 | 63 | 46 | 8 |
| MDJ-6 | 21 | 3 | 1 | 3 | 4 | 3 | 2 | 1 | 1 |
| MDJ-8 | 1150 | 25 | 24 | 134 | 283 | 43 | 4 | 8 | 238 |
| MDJ-9 | 1151 | 258 | 10 | 111 | 139 | 2 | 9 | 6 | 1 |
| ZD4 | 282 | 6 | 5 | 1 | 33 | 24 | 4 | 7 | 19 |
| ZD3 | 1128 | 12 | 5 | 72 | 13 | 1 | 46 | 34 | 9 |
| ZD2 | 32 | 22 | 5 | 17 | 21 | 15 | 16 | 7 | 19 |
| HLBR1 | 1133 | 129 | 5 | 230 | 1 | 8 | 19 | 4 | 1 |
| HLBR2 | 1133 | 129 | 5 | 230 | 1 | 8 | 19 | 4 | 1 |
| HLBR3 | 1135 | 252 | 160 | 151 | 279 | 136 | 174 | 18 | 62 |
| HLBR4 | 1136 | 251 | 5 | 1 | 11 | 2 | 4 | 7 | 8 |
| SJZ1 | 119 | 3 | 24 | 1 | 46 | 3 | 2 | 4 | 1 |
| SJZ2 | 119 | 3 | 24 | 1 | 46 | 3 | 2 | 4 | 1 |
| SJZ4 | 1143 | 256 | 4 | 46 | 148 | 2 | 27 | 22 | 41 |
| SJZ-6 | 531 | 43 | 4 | 46 | 102 | 2 | 27 | 22 | 41 |
| SJZ-7 | 1125 | 64 | 3 | 63 | 4 | 2 | 2 | 1 | 11 |
| SC2-1 | 437 | 20 | 63 | 46 | 94 | 45 | 82 | 48 | 61 |
| SC4-1 | 597 | 19 | 85 | 1 | 4 | 5 | 2 | 4 | 1 |
| SC5-1 | 611 | 4 | 24 | 47 | 4 | 3 | 2 | 1 | 1 |

*Red indicates new sequence types (STs), and blue indicates new alleles.
